# Association Between Apolipoprotein E Polymorphism and Subclinic Atherosclerosis in Patients with Type 1 Diabetes Mellitus

**DOI:** 10.4274/jcrpe.521

**Published:** 2012-03-08

**Authors:** Mehmet Emre Atabek, Yusuf Özkul, Beray Selver Eklioğlu, Selim Kurtoğlu, Murat Baykara

**Affiliations:** 1 Selçuk University, Meram Faculty of Medicine, Department of Pediatric Endocrinology, Konya, Turkey; 2 Erciyes University, School of Medicine, Department of Genetics, Kayseri, Turkey; 3 Erciyes University, School of Medicine, Department of Pediatric Endocrinology, Kayseri, Turkey; 4 Erciyes University, School of Medicine, Department of Radiology, Kayseri, Turkey; +90 332 223 63 50 berayselver@hotmail.comSelçuk University Meram Faculty of Medicine, Department of Pediatric Endocrinology, Konya, Turkey

**Keywords:** Atherosclerosis, apolipoprotein E, carotid artery intima-media thickness, type 1 diabetes

## Abstract

**Objective** The most important cause of morbidity and mortality in type 1 diabetes mellitus (DM) is atherosclerosis. Apolipoprotein E (Apo E) polymorphism is accused of being the genetic risk factor for atherosclerosis. The aim of the present study was to determine which Apo E polymorphism was related to atherosclerosis in patients with type 1 DM.

**Methods:** Seventy-four patients with type 1 DM were enrolled in the study. Age, diabetes duration, daily insulin dose, microalbuminuria, and major cardiovascular risk factors including anthropometric and metabolic parameters were assessed in each patient. Non-invasive ultrasonographic measurements were also performed. For determination of Apo E genotype, DNA was extracted from venous blood from all subjects using standard methods. Apo E genotyping was performed using a PCR–restriction fragment-length polymorphism assay.

**Results:** Systolic blood pressure and carotid artery intima-media thickness (CA-IMT) were increased in subjects with E4/E4 polymorphism. According to univariate analysis, when adjusted for all risk factors, genotypes did not differ for total cholesterol, high-density lipoprotein cholesterol, low-density lipoprotein cholesterol and triglycerides (p>0.05). However, E3/E3, E3/E4 and E4/E4 genotypes were found to be associated with an increase in CA-IMT (p<0.001).

**Conclusions:** Our results suggest that the polymorphism associated with atherosclerosis in type1 DM is Apo E4/E4.

**Conflict of interest:**None declared.

## INTRODUCTION

Diabetes mellitus (DM) is a complex disorder characterized by hyperglycemia. Cardiovascular disease is frequent in type 1 DM. The most important cause of morbidity and mortality in type 1 DM is atherosclerosis (1).

It has recently been shown that ultrasonography is a powerful non-invasive tool for evaluating early atherosclerotic lesions in the carotid artery (2,3,4). Increased carotid artery intima-media thickness (CA-IMT) is significantly related to cardiovascular risk factors and to carotid plaque, which is a more advanced atherosclerotic lesion. Increased CA-IMT may be observed even in adolescents with type 1 DM (5).

Apolipoprotein E (Apo E) polymorphism has been accused as being the responsible factor for development of atherosclerosis. Apo E plays a major role in lipid metabolism and has a high affinity for low-density lipoprotein (LDL) receptors (6). It has three common isoforms (E2, E3, and E4) encoded by three alleles (e2, e3, e4) in exon 4 of the Apo E gene. Apo E3 is the most common form (7). 

The aim of our present study was to assess the atherosclerosis indices and CA-IMT in patients with type 1 DM and also to investigate the relationship between Apo E polymorphism and these factors. Especially, we aimed to find which Apo E polymorphism is associated with atherosclerosis.

## METHODS

This study was performed in the Pediatric Endocrinology Department at Erciyes University School of Medicine. The study was approved by the local ethics committee of Erciyes University and was conducted in accordance with the guidelines proposed in the Declaration of Helsinki. Signed informed consent was obtained from the parents.

Seventy-four patients (38 female, 36 male) with type 1 DM participated in this study. Sex, age, anthropometric parameters, blood pressure (BP), lipid profiles, daily insulin dose, urinary albumin excretion, glycated hemoglobin (HbA1c) level, common carotid artery functions and Apo E genotype were determined in each patient. 

Patients under 19 years of age, with type 1 DM, using insulin therapy, and who had been followed for at least 1 year were planned to be included in the study; thus, 38 female and 36 male patients aged between 8 and 18 were recruited. None of the patients had diseases such as hypertension, hyperlipidemia and other cardiovascular diseases known to affect the common carotid artery functions, and no patient was under any medication other than antidiabetic drugs. None of the patients had ever smoked. 

Twenty four-hour urine samples were collected and analyzed for presence of albuminuria after excluding proteinuria due to urinary tract infection. Microalbuminuria was defined as a 24-h urinary albumin excretion of >30 mg. A urinary albumin excretion exceeding 300mg/24h in at least two urine samples evaluated within a 12-week interval was accepted to indicate clinical nephropathy. None of the patients were diagnosed to have renal disease unrelated to diabetes during their follow-up.

Hyperlipidemia was defined as a serum lipid level higher than the 95^th^ percentile for age and sex. BP was measured with a standard mercury sphygmomanometer after the subjects had rested for at least 10 min. Subjects were considered as hypertensive if their systolic BP and/or their diastolic BP were above the 95^th^ percentile for age and sex, or if they were receiving antihypertensive treatment. Anthropometric measurements were performed by the same clinician in all subjects. Height was measured using a wall-mounted stadiometer and weight was determined using a balance scale with the subject dressed only in light underwear and wearing no shoes. Body mass index (BMI) was calculated as the ratio of weight (kg) to the square of height (m)2. Hip circumference and waist circumference were measured and recorded.

Blood samples were collected after a minimum eight hours of fasting. Serum lipid concentrations and urine microalbumin levels were assayed using the Konelab 60i analyser (Konelab, Espoo, Finland). Plasma HbA1c concentrations were assayed by automated high-performance liquid chromatography (reference rate <6%).

**Determination of Apo E polymorphism **

To perform Apo E genotyping assays, venous blood was collected from all subjects in sterile EDTA 4-mL tubes. Genomic DNA was extracted from whole blood using High Pure PCR Template Preparation kits (Roche Diagnostics, GmbH Mannheim, Germany). Apo E polymorphism was determined by quantitative real-time reverse transcription-polymerase chain reaction, which is a well-established and validated method (LightCycler Apo E Mutation Detection Kit; Roche Diagnostics) (Ballerini et al 2002). A 265-bp fragment of Apo E gene was amplified with specific primers from human genomic DNA. The amplicon was detected by fluorescence using a pair of hybridization probes. The hybridization probes were also used to determine the genotype by performing melting curve analysis after the amplification cycles were completed.

**Common carotid artery ultrasonography**

All examinations were done by the same sonographer, who was blinded to the participant’s case status and risk factor levels. High-resolution B-mode ultrasonography of the right common carotid artery was performed with SSA-370A Power Vision 6000 Digital Ultrasound System equipped with a 7.5 MHz linear transducer (Toshiba Corporation, Tokyo). Subjects were kept in a quiet, dark, temperature-controlled room and rested in supine position for about 15-20 minutes. They were examined in the supine position with the head slightly extended and turned slightly to the left. Longitudinal images of the common carotid artery were obtained by combined B-mode and color Doppler ultrasound examinations. IMT of the posterior (far) wall of the common carotid artery was measured with the electronic calipers of the machines, as described by Pignoli et al (8). On a longitudinal, 2-dimensional ultrasound image of the carotid artery, images of the posterior wall of the carotid artery are displayed as 2 bright white lines separated by a hypoechoic space. The distance between the leading edge of the first bright line of the far wall and the leading edge of the second bright line indicates the IMT. The IMT was measured during end diastole. The IMT measurements were performed on-line. The mean IMT was calculated by taking the average value of three measurements obtained from the common carotid artery; 20 mm below the carotid bulb.

## STATISTICS

Data analysis was performed using SPSS for Windows 11.5 package program. Kolmogorov Smirnov test was used to determine whether continuous variables were normally distributed. Descriptive statistics for continuous measurement variables were shown as mean±standard deviation or median (interquartile width). Nominal variables were presented as number of cases as well as in percentages (%).

Significance of difference in terms of averages was evaluated between the groups. When the number of independent groups was limited to two, the Student’s t-test was used. The significance of the differences between more than two groups was evaluated in the parametric one-way analysis of variance (one-way ANOVA). Significance of differences in terms of medians was evaluated between the groups. When the number of independent groups was two, the Mann-Whitney U-test was used. The significance of the differences between more than two groups was evaluated applying the nonparametric one-way ANOVA (Kruskal-Wallis). In instances of significant results with the Kruskal-Wallis test, nonparametric multiple comparison tests were used to identify the conditions that caused the difference.

Nominal variables were evaluated with Pearson's chi-square test. Statistically significant correlations between continuous variables were assessed by the Spearman correlation test. All possible risk factors thought to influence the atherosclerosis indicators were adjusted. The multivariate linear regression analysis was applied to detect any statistically significant difference between genotypes. Regression coefficient for each variable was calculated with 95% confidence interval. LDL cholesterol, high-density lipoprotein (HDL) cholesterol and CA-IMT variables were not normally distributed and logarithmic transformation was performed for the linear regression analysis.

A p-value of less than 0.05 was considered statistically significant for the results.

## RESULTS

The clinical and laboratory characteristics and CA-IMT measurements of the patients according to Apo E genotypes are shown in Tables 1 and 2. Systolic BP, triglyceride level and CA-IMT were significantly different between genotypes. Systolic BP and CA-IMT were higher in E4/E4 genotype, while triglyceride level was higher in E3/E4 genotype.

There were no significant differences in CA-IMT measurements, total cholesterol, triglyceride and HDL levels between girls and boys. The p-values were 0.106, 0.105, 0.799 and 0.559, respectively.

The correlations between clinical characteristics and total cholesterol, HDL cholesterol, LDL cholesterol.

Correlation coefficients, significance levels between CA-IMT measurement and clinical characteristics are shown in Table 3. CA-IMT was positively correlated with age, BMI, waist-to-hip ratio and systolic BP.

According to univariate analysis, when adjusting for all risk factors, genotypes did not differ for total cholesterol, HDL cholesterol, LDL cholesterol and triglycerides (p>0.05). However, E3/E3, E3/E4 and E4/E4 genotypes were found to be associated with increased CA-IMT (p<0.001).

## DISCUSSION

Type 1 DM is associated with a two- to fourfold higher risk of coronary artery disease (9). The increase of cardiovascular risk in this population cannot be explained only by traditional risk factors.

Studies on potential gene polymorphisms that can directly affect the development of atherosclerosis are rare (10). Apo E gene polymorphism is thought to cause an alteration in lipoprotein metabolism. Apo E-deficient mice have been reported to develop severe dyslipidemia and atherosclerotic lesions (11). The E4 allele has been found to be related with LDL-cholesterol levels. However, the effects of Apo E polymorphism on triglyceride and HDL cholesterol levels have not been resolved (12).

Studies have shown that increased CA-IMT is a predictor of atherosclerosis. Associations between Apo E alleles and cardiovascular risk have also been reported (13,14). Wohlin et al (15) found increased CA-IMT in Apo E4 homozygous genotype. In the ARIC and CUDAS studies, E4/E4-homozygous men were reported to have a significantly increased CA-IMT (16,17). In the Rotterdam study, men with the e4/e4 genotype had slightly, but not significantly increased CA-IMT (18). These studies were performed in elderly persons and only in men. To our knowledge, there are no studies assessing the relationship between CA-IMT and Apo E polymorphism in children with type 1 DM. In our study, we found higher CA-IMT in the E4/E4 homozygous genotype subjects.

It has been reported that the E4 and E3 alleles increase plasma cholesterol concentration, while E2 alleles have an opposite effect (3,19). It was also reported that subjects with E4 allele had higher lipid levels, especially LDL cholesterol levels (20). Tascilar et al (20) and Fernandez et al (21) showed that the E4 allele was associated with elevated LDL cholesterol. Bennet et al (22) reported that Apo E genotypes were related to LDL levels and coronary risk. E2 carriers were found to have 20% reduced coronary disease risk, while E4 carriers had increased risk. In this study, we found no significant association between LDL cholesterol, HDL cholesterol, total cholesterol and Apo E genotypes. Only triglyceride levels were significantly different between Apo E genotypes and higher in E3/E4 allele. We think that these results may be associated with the smallness of our sample.

In some populations, a stronger association between Apo E 4 allele and coronary artery disease was described for men (23). However, a meta-analysis study revealed that Apo E genotypes were not related to gender (22). No gender difference was found in the present study.

Type 1 DM is an important risk factor for the development of cardiovascular diseases (24). Carotid artery stiffness and IMT measured by ultrasonography are correlated with atherosclerosis and cardiovascular diseases in patients with type 1 DM (25). Several groups have demonstrated that patients with type 1 DM have higher mean CA-IMT. Increased CA-IMT in children with type 1 DM may be an early step in the development of atherosclerosis. Yavuz et al (26) and Gunczler et al (27) found no increase of CA-IMT in children with type 1 DM. However, Yamasaki et al (28) Jarvisalo et al (29) and Vastagh I et al (30) reported that CA-IMT values were significantly higher in children with type 1 DM. Jarvisalo et al (29) also showed that type 1 DM was an independent risk factor for high CA-IMT. Similarly, we demonstrated increased CA-IMT in type 1 DM previously (31,32).

It is known that familial hypercholesterolemia and borderline hypertension are related to high IMT (29,33). Also, BMI was reported to be correlated with CA-IMT (34). Childhood obesity seems to contribute to the development and progression of early atherosclerosis. Zhu et al (35) found higher CA-IMT in obese Chinese school children. We also found that CA-IMT positively correlated with BMI, waist-to-hip ratio, and systolic BP in our study.

Our study demonstrates the association of Apo E4/E4 polymorphism with CA-IMT and coronary artery disease in type 1 diabetes patients and this association is independent of anthropometric and metabolic measurements. Apo E4/E4 allele might be the major genetic risk factor for increased CA-IMT and atherosclerosis in children with type 1 DM.

## Figures and Tables

**Table 1:**
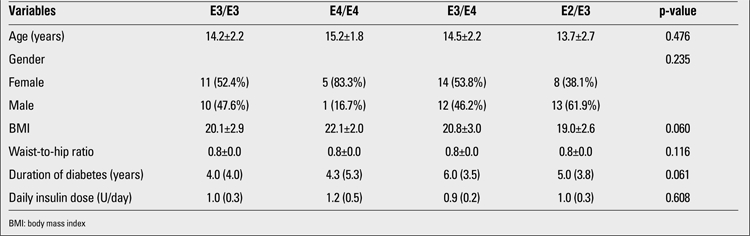
Clinical findings according to genotypes

**Table 2:**
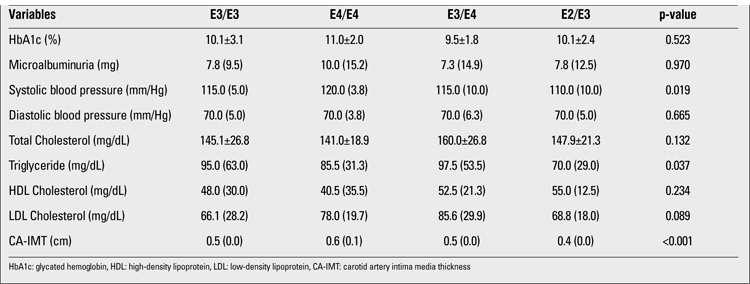
Laboratory findings according to genotypes

**Table 3:**
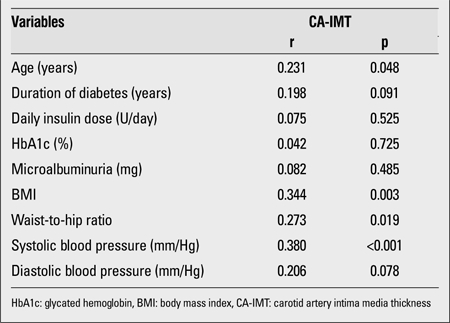
Correlation coefficients, significance levels between CA-IMT measurements and clinical characteristics 14-20
